# 2311. Long-term prognosis and risk factors for overall mortality in patients with progressive multifocal leukoencephalopathy

**DOI:** 10.1093/ofid/ofac492.143

**Published:** 2022-12-15

**Authors:** Jinnam Kim, Chang Hyup Kim, Jung Ah Lee, Se Ju Lee, Ki Hyun Lee, Jung Ho Kim, Jin Young Ahn, Su Jin Jeong, Nam Su Ku, Jun Yong Choi, Joon-sup Yeom, Young Goo Song

**Affiliations:** Yonsei University College of Medicine, Seoul, Seoul-t'ukpyolsi, Republic of Korea; Yonsei University College of Medicine, Seoul, Seoul-t'ukpyolsi, Republic of Korea; Yonsei University College of Medicine, Seoul, Seoul-t'ukpyolsi, Republic of Korea; Inha University College of Medicine, Seoul, Seoul-t'ukpyolsi, Republic of Korea; Yonsei University College of Medicine, Seoul, Seoul-t'ukpyolsi, Republic of Korea; Yonsei University College of Medicine, Seoul, Seoul-t'ukpyolsi, Republic of Korea; Yonsei University College of Medicine, Seoul, Seoul-t'ukpyolsi, Republic of Korea; Yonsei University College of Medicine, Seoul, Seoul-t'ukpyolsi, Republic of Korea; Division of Infectious Diseases, Department of Internal Medicine, Yonsei University College of Medicine, Seoul, Seoul-t'ukpyolsi, Republic of Korea; Yonsei University College of Medicine, Seoul, Seoul-t'ukpyolsi, Republic of Korea; Division of Infectious Diseases, Department of Internal Medicine, Yonsei University College of Medicine, Seoul, Seoul-t'ukpyolsi, Republic of Korea; Yonsei University College of Medicine, Seoul, Seoul-t'ukpyolsi, Republic of Korea

## Abstract

**Background:**

Progressive multifocal leukoencephalopathy (PML) is a demyelinating disease of the central nervous system (CNS) caused by reactivation of JC virus, almost in patients with immunosuppressive conditions. PML is a fatal infection with a reported 3-month mortality rate of 30–50% and a 2-month mortality rate of up to 90% in the non-human immunodeficiency virus (HIV) population. Despite high mortality, studies on PML are still lacking due to its low prevalence and incidence. Therefore, this study aimed to figure out long-term prognosis of PML and prognostic factors for mortality through a long observation period.

**Methods:**

We retrospectively reviewed 68 PML patients with admitted to two tertiary hospitals in South Korea from 1999 to 2021. A total of 47 PML patients were finally enrolled after exclusion. The primary endpoint was long-term overall mortality. For survival analysis, Kaplan-Meier curve and Cox proportional hazards model were used. Each patient was followed up until death or until the end of the study period, whichever came first.

**Results:**

The median follow-up duration was 20 (interquartile range [IQR], 3–79) months. The median age was 46 years, 27 (57.4%) were diagnosed with HIV, 19 (40.4%) were using immunosuppressive drugs. The median last follow-up modified Rankin Scale (mRS) was higher in non-HIV PML patients group (5 [IQR, 4–6] vs 4 [IQR, 2–5], p=0.020). The median survival duration was 184 (IQR 74–1,566) days in the non-HIV group and 1,564 (IQR 254–3,444) days in the HIV group. The overall mortality rate of PML patients was significantly higher in non-HIV group (80.0% vs 40.7%, p=0.007), also confirmed by the Kaplan-Meier curve and log-rank test (p=0.007). Initial mRS (HR 1.685, 95% CI: 1.028–2.762, p=0.038), HIV patients with highly active antiretroviral therapy (HAART) (HR 0.374, 95% CI: 0.172–0.815, p=0.013) had a significant effect on overall mortality.

**Conclusion:**

With the widespread adoption of HAART, the survival duration of HIV patients with PML has been extended, but the mortality rate is still high. Also, the prognosis for PML in non-HIV patients is still frustrated. Initial mRS is a significant risk factor for long-term overall mortality in PML patients. Early detection of PML and early initiation of HAART in HIV patients may improve the patient's prognosis.

**Disclosures:**

**All Authors**: No reported disclosures.

